# *NAMPT* gene rs2058539 variant is a risk factor for nonalcoholic fatty liver disease

**DOI:** 10.1590/1806-9282.20230188

**Published:** 2024-07-19

**Authors:** Shadi Nouri, Mahsa Navari, Fatemeh Zarei, Mitra Rostami, Touraj Mahmoudi, Gholamreza Rezamand, Asadollah Asadi, Hossein Nobakht, Reza Dabiri, Seidamir Pasha Tabaeian

**Affiliations:** 1 Arak University of Medical Sciences, School of Medicine, Department of Radiology – Arak, Iran.; 2 Shahid Beheshti University of Medical Sciences, Research Institute for Gastroenterology and Liver Diseases, Gastroenterology and Liver Diseases Research Center – Tehran, Iran.; 3 Università degli Studi di Milano, Department of Pharmaceutical Sciences – Milan, Italy.; 4 Iran University of Medical Sciences, School of Medicine, Department of Internal Medicine – Tehran, Iran.; 5 Iran University of Medical Sciences, Colorectal Research Center – Tehran, Iran.; 6 University of Mohaghegh Ardabili, Faculty of Science, Department of Biology – Ardabil, Iran.; 7 Semnan University of Medical Sciences, Department of Internal Medicine – Semnan, Iran.

**Keywords:** NAFLD, NAMPT, Variant, Visfatin

## Abstract

**OBJECTIVE:**

Nonalcoholic fatty liver disease is a chronic liver disease and a growing global epidemic. The aim of this study was to investigate the association between a visfatin gene (*NAMPT*) variant and nonalcoholic fatty liver disease, owing to the connection between this disease and insulin resistance, obesity, inflammation, and oxidative stress, and the role of visfatin in these metabolic disorders.

**METHODS:**

In the present case-control study, we enrolled 312 genetically unrelated individuals, including 154 patients with biopsy-proven nonalcoholic fatty liver disease and 158 controls. The rs2058539 polymorphism of *NAMPT* gene was genotyped using the PCR-RFLP method.

**RESULTS:**

Genotype and allele distributions of *NAMPT* gene rs2058539 polymorphism conformed to the Hardy-Weinberg equilibrium both in the case and control groups (p>0.05). The distribution of *NAMPT* rs2058539 genotypes and alleles differed significantly between the cases with nonalcoholic fatty liver disease and controls. The "CC" genotype of the *NAMPT* rs2058539 compared with "AA" genotype was associated with a 2.5-fold increased risk of nonalcoholic fatty liver disease after adjustment for confounding factors [p=0.034; odds ratio (OR)=2.52, 95% confidence interval (CI)=1.36–4.37]. Moreover, the *NAMPT* rs2058539 "C" allele was significantly overrepresented in the nonalcoholic fatty liver disease patients than controls (p=0.022; OR=1.77, 95%CI=1.14–2.31).

**CONCLUSION:**

Our findings indicated for the first time that the *NAMPT* rs2058539 "CC" genotype is a marker of increased nonalcoholic fatty liver disease susceptibility; however, it needs to be supported by further investigations in other populations.

## INTRODUCTION

Nonalcoholic fatty liver disease (NAFLD) as the most common cause of chronic liver disease is a multifactorial disorder in which liver fat exceeds 5% of hepatocytes in the absence of the secondary factors of hepatic fat aggregation or significant alcohol consumption. A growing body of research shows that the prevalence of NAFLD which is currently around 23%–25% of adults in the world will probably increase more owing to the ongoing obesity epidemic. NAFLD patients are more predisposed to have insulin resistance (IR), type 2 diabetes (T2D), and obesity. IR also affects the rate of elevation of serum liver enzymes in NAFLD, and the rate is higher in NAFLD patients with IR than those without IR^1-3^. Interestingly, significant associations between insulin receptor (*INSR*), insulin-like growth factor 1 (*IGF1*), insulin-like growth factor-binding protein 3 (*IGFBP3*), and visfatin (*NAMPT*) gene polymorphisms, and the risk of NAFLD have been discovered^4-7^.

Nicotinamide phosphoribosyltransferase (NAMPT) or visfatin which is expressed in a variety of tissues including hepatocytes and adipocytes is the product of the *NAMPT* gene. NAMPT plays a role in the production of nicotinamide adenine dinucleotide. In addition to its aforementioned intracellular enzymatic activity, visfatin also has an extracellular action as a cytokine mainly in inflammation. It appears that visfatin is involved in the development of NAFLD by modulating disorders, including obesity, IR, inflammation, and oxidative stress. Previous reports have indicated positive correlations between circulating visfatin level and IR^8^, T2D^9^, triglycerides^10^, body mass index (BMI)^8,9^, metabolic syndrome^9^, liver enzymes^10^, and NAFLD^11^. Significant associations between *NAMPT* gene variants and the expression of *NAMPT* gene^12^ and circulating level of visfatin^10^ have also been found. Thus, the aim of the current research was to explore the possible contribution of *NAMPT* gene to NAFLD pathogenesis. The rs2058539 variant of *NAMPT* gene was selected based on high degree of heterozygosity and its usage in previous research.

## METHODS

### Study population

After informed consent, 154 patients with biopsy-proven NAFLD (age range, 31–87 years) and 158 controls (age range, 31–81 years) were enrolled in the present retrospective case-control study. NAFLD patients were enrolled after fatty liver diagnosis which in turn was defined by (a) ultrasonographic confirmation of fatty liver, (b) having high circulating levels of liver enzymes, (c) excluding subjects with other causes of liver disease like Wilson's disease, alpha-1 antitrypsin deficiency, viral hepatitis, and alcohol use more than 70 g/week in women or more than 140 g/week in men, and (d) liver biopsy evidence of NAFLD by an experienced pathologist who examined the liver biopsy samples using the Brunt's criteria. Steatosis and necroinflammation grades were 0–3 and fibrosis stages were 0–4. On the contrary, the control group was recruited from the research staff of the Institute. Those who were free of elevated liver enzymes and viral hepatitis infection (examined by blood tests), had no liver steatosis (examined by abdominal ultrasonography), and were not alcoholic or on regular medications were enrolled as controls. Self-report questionnaires were used for collecting the study population characteristics. This study complied with the principles of the Declaration of Helsinki and was performed according to the Institute's Ethics Committee approval^13,14^.

### Genotyping

Blood samples were collected in EDTA vials, and genomic DNA was purified from the white blood cells using phenol chloroform extraction and ethanol precipitation protocol and then stored at −20°C until use. The genotypes of the *NAMPT* gene rs2058539 variant were determined using PCR-RFLP. Genomic DNA was amplified using the primers: 5’-ATTAACTTTGGTATTCTTGCC-3’ and 5’-TAGCCAGTTTTACCTTGAAGAC-3’ to detect the genotypes of the *NAMPT* rs2058539 polymorphism. PCR was carried out with an initial denaturation at 95°C for 10 min, followed by 35 cycles of denaturation at 95°C for 45 s, annealing at 61°C for 45 s, and extension at 72°C for 45 s. The final extension was at 72°C for 10 min. The PCR products were digested with HphI (Fermentas, Leon-Rot, Germany) in a water bath at 37°C overnight, subjected to agarose gel electrophoresis, and visualized through ethidium bromide (0.5 μg/mL) staining using a UV transilluminator^15^. The "C" allele of the *NAMPT* rs2058539 had bands of 216 bp and 134 bp, whereas its "A" allele had a band of 350 bp, thus an individual with band(s) at 216 and 134 bp, at 350 bp only, and at 350, 216, and 134 bp was defined as CC homozygotic genotype, AA homozygotic genotype, and AC heterozygotic genotype, respectively. Genotyping of 20% of all the samples was performed twice by different laboratory personnel; the reproducibility was 100%.

### Statistical analysis

To perform statistical analyses, the SPSS software package for Windows, version 25.0 (SPSS Inc. Chicago, IL, USA), was used. To examine the normality of distribution of continuous variables, the Kolmogorov-Smirnov goodness-of-fit test was used. The demographic, anthropometric, clinical, and biochemical features of the patients with NAFLD were compared with those found in the controls by Student's unpaired t-test or chi-square (χ^2^) test as appropriate. Hardy-Weinberg equilibrium (HWE) for the *NAMPT* gene rs2058539 polymorphism in the control and case groups was also separately analyzed by χ^2^ test with 1 degree of freedom, comparing the observed genotype frequencies with those expected. The differences in allele frequencies between the NAFLD and control groups were also assessed using χ^2^ test. To appraise the association between the genotype frequencies and NAFLD and to adjust confounding factors, we employed logistic regression analysis. The odds ratios (OR) and their respective 95% confidence intervals (95%CI) were used as the measure of strength for the associations. Differences in biochemical parameters among the different *NAMPT* genotypes were tested by analysis of variance (ANOVA) and analysis of covariance (ANCOVA) when appropriate. A value of p<0.05 was taken as a statistically significant difference.

## RESULTS

Table 1 presents the demographic, anthropometric, clinical, and biochemical data of the NAFLD patients and controls. The cases were more likely to be male (p<0.001) and smoker (p=0.011) and had higher age (P<0.001), BMI (P<0.001), systolic blood pressure (p<0.001), diastolic blood pressure (p<0.001), AST (p<0.001), ALT (p<0.001), and GGT (p<0.001) than the controls.

**Table 1:** General characteristics of the study groups.

Variables^a^		Controls (n=158)	Cases with nonalcoholic fatty liver disease (n=154)	p-value
Age (years)		28.9 (7.7)	39.1 (9.1)	<0.001
BMI (kg/m^2^)		23.3 (3.5)	29.5 (5.2)	<0.001
Sex				
	Male	83 (52.5)	114 (74.0)	
	Female	75 (47.5)	40 (26.0)	<0.001
Smoking history				
	No	143 (90.5)	115 (74.7)	
	Former	10 (6.3)	20 (13.0)	
	Current	5 (3.2)	19 (12.3)	0.011
	SBP (mmHg)	114.8 (13.2)	123.7 (15.2)	<0.001
	DBP (mmHg)	69.4 (8.3)	75.0 (9.6)	<0.001
	AST (IU/L)	19.6 (7.3)	39.8 (17.4)	<0.001
	ALT (IU/L)	19.4 (10.6)	72.2 (41.3)	<0.001
	GGT (IU/L)	18.3 (8.5)	58.8 (31.7)	<0.001
Steatosis				
	Grade 0		-	
	Grade 1		40 (26.0)	
	Grade 2		83 (53.9)	
	Grade 3		31 (20.1)	
Necroinflammation				
	Grade 0		48 (31.2)	
	Grade 1		58 (37.7)	
	Grade 2		46 (29.8)	
	Grade 3		2 (1.3)	
Fibrosis				
	Stage 0		88 (57.2)	
	Stage 1		59 (38.3)	
	Stage 2		7 (4.5)	
	Stage 3		-	
	Stage 4		-	

a Variables presented as mean (SD) or number (%); BMI: body mass index, SBP: systolic blood pressure; DBP: diastolic blood pressure, AST: aspartate aminotransferase; ALT, alanine aminotransferase; GGT: gamma glutamyl transferase.

Genotype and allele distributions of the *NAMPT* rs2058539 polymorphism conformed to HWE test both in the case and control populations (p>0.05). This implies that a representative sample population was used in this study. Analysis of this SNP revealed a significant difference between the cases and the controls (Table 2). The "CC" genotype of the *NAMPT* rs2058539 compared with "AA" genotype was associated with a 2.5-fold increased risk of NAFLD after adjustment for confounding factors (p=0.034; OR=2.52, 95%CI=1.36–4.37). In addition, the *NAMPT* rs2058539 "C" allele was significantly overrepresented in the NAFLD patients than the controls (p=0.022; OR=1.77, 95%CI=1.14–2.31).

**Table 2:** Distribution of the visfatin gene (*NAMPT*) rs2058539 variant in the cases with biopsy-proven nonalcoholic fatty liver and the controls^a^.

Gene (Variant)	Controls (n=156)	Cases (n=152)	OR (95% CI) p-value^b^
NAMPT (rs2058539)^c^			
Genotype-wise comparison			
AA	81 (51.9)	61 (40.1)	1.0 (reference)
AC	62 (39.8)	64 (42.1)	1.92 (0.67–5.51)0.225
CC	13 (8.3)	27 (17.8)	2.52 (1.36–4.37)0.034
AC and CC	75 (48.1)	91 (59.9)	2.29 (0.84–6.25)0.107
CC versus others	13 (8.3)	27 (17.8)	3.82 (0.73–8.09)0.081
Allele-wise comparison				
A	224 (71.8)	186 (61.2)	1.0 (reference)
C	88 (28.2)	118 (38.8)	1.77 (1.14–2.31)0.022

a Variables presented as number (%).

b Adjusted for age, body mass index (BMI), sex, smoking status, systolic blood pressure (SBP), and diastolic blood pressure (DBP) in genotype-wise comparisons.

c Distribution of the *NAMPT* gene rs2058539 variant in 156 controls and 152 patients.

Table 3 shows the association between the rs2058539 variant of *NAMPT* gene and anthropometric, biochemical, and pathological parameters in 152 patients with biopsy-proven NAFLD; no significant association was found.

**Table 3:** Association between *NAMPT* gene rs2058539 polymorphism and different variables in 152 patients with biopsy-proven nonalcoholic fatty liver.

Variables^a^	AA+AC	CC	p-value
Number	125	27	
BMI (kg/m^2^)	29.8 (5.3)	27.9 (5.1)	0.754
AST (IU/L)	39.6 (17.3)	41.0 (16.8)	0.572
ALT (IU/L)	72.5 (41.8)	70.9 (40.2)	0.629
GGT (IU/L)	57.0 (30.2)	67.1 (33.5)	0.197
SBP (mmHg)	122.8 (14.3)	127.6 (15.9)	0.466
DBP (mmHg)	74.5 (9.2)	77.3 (9.8)	0.523
Hypertension	30 (24.0)	8 (29.6)	0.206
Diabetes	26 (20.8)	6 (22.2)	0.549
Steatosis, Grade 3	24 (19.2)	7 (25.9)	0.294
Necroinflammation, Grades 2, 3	40 (32.0)	8 (29.6)	0.601
Fibrosis, Stages 1, 2	52 (42.4)	12 (44.4)	0.739

a Variables presented as mean (SD) or number (%); BMI: body mass index, SBP: systolic blood pressure; DBP: diastolic blood pressure, AST: aspartate aminotransferase; ALT: alanine aminotransferase, GGT: gamma glutamyl transferase.

## DISCUSSION

Genes involved in glucose metabolism, IR, fatty acid metabolism, obesity, oxidative stress, inflammation, and fibrosis development are among the candidate genes for NAFLD. Interestingly, visfatin participates in many of these pathways, hence it is not unreasonable to presume that visfatin and its gene (*NAMPT*) play a role in NAFLD pathogenesis. The human *NAMPT* gene that consists of 11 exons is a highly polymorphic gene and has a wide variety of biological functions, so any defects in it may lead to IR, obesity, and inflammation that are implicated in the pathogenesis of NAFLD^16^. In this research, we found that the *NAMPT* rs2058539 "CC" genotype in comparison to the "AA" genotype increases the risk of NAFLD more than 2.5-fold. Consistently, the *NAMPT* rs2058539 "C" allele was more frequent in the cases with NAFLD too. Of note, RNA splicing and protein expression may be influenced by alterations in the intronic sequences. Perhaps, the *NAMPT* rs2058539 variant (located in intron 1) per se not to be functional; instead, it could be in linkage disequilibrium with another functional variant of the *NAMPT* gene. Consistently, Chang et al. have shown that *NAMPT* rs2058539 and *NAMPT* rs61330082 are in a linkage disequilibrium block, and the latter SNP is associated with the transcription rate of *NAMPT* gene, and serum levels of NAMPT, total cholesterol, triglyceride, and C-reactive protein, as well as cardiovascular diseases^8^. The other possibility is that the rs2058539 "C" allele might be more stable and translates more efficiently into visfatin, which in turn finally increases the risk of NAFLD. Prior investigations have also shown significant associations between *NAMPT* gene variants and its transcription rate^12^, and circulating levels of visfatin^10^, triglyceride^10^, glucose, and insulin^10^. There have been associations between *NAMPT* gene polymorphisms and risk of obesity^16^ and T2DM^17^ too. Visfatin, an insulin-mimetic adipokine, can link IR with obesity. NAMPT is involved in glucose homeostasis and the regulation of glucose-stimulated insulin secretion in pancreatic β cells through NAD biosynthesis, and it induces IR. Interestingly, glucose and insulin regulate the release of visfatin^8,9,18^. Consistently, NAMPT levels were positively associated with triglyceride^10^, BMI^8,9^, IR^8^, T2D^9^, metabolic syndrome^9^, liver enzymes^10^, and NAFLD^11^. There is also more evidence that supports the hypothesis that visfatin plays a role in NAFLD. Visfatin is a proinflammatory cytokine and its level is positively associated with portal inflammation^9^. NAMPT induces the expression of pro-inflammatory cytokines, including TNF-α, IL-1β, and IL-6 via the STAT3 and NF-κB signaling pathways^18^. The NF-κB pathway has a key role in inflammation, and visfatin induces its activation. The injection of visfatin in mice aggravates inflammation, hepatic steatosis, and fibrosis, and increases oxidative stress and plasma levels of liver enzymes^19^. Visfatin also regulates the expression of some important microRNAs (miRNAs), including miR-146a, miR-155, and miR-181a that participate in inflammation and the activation of the immune system cells. MiRNAs are non-coding and single-stranded RNA molecules containing 22–25 nucleotides, which act in post-transcriptional regulation of gene expression^20,21^. From the above accumulating evidence, it appears plausible to assume that visfatin and its gene, *NAMPT*, may be involved in the development of NAFLD.

This study had strengths and limitations. First, the sample size of our study was modest due to using liver biopsy. Hence, performing sub-analyses was unreasonable. Second, owing to the fund limitations, we were unable to evaluate serum levels of visfatin. However, the design of this study was good and, more importantly, we employed liver biopsy as the gold standard method for confirming NAFLD diagnosis. Moreover, our report presented some novel and interesting findings.

## CONCLUSION

Our findings revealed a role for the *NAMPT* rs2058539 gene variant in the pathogenesis of NAFLD: the carriers of *NAMPT* rs2058539 "CC" genotype had a 2.5-fold increased risk for NAFLD. This observation is made for the first time and it supports previous literature; however, further research is needed to confirm it.

## INSTITUTE WHERE THE RESEARCH WAS CONDUCTED

Research Institute for Gastroenterology and Liver Diseases, Shahid Beheshti University of Medical Sciences, Velenjak, Shahid Chamran Highway, Tehran, Iran.
